# Traumatic Craniocervical Dissociation in Patients with Congenital Assimilation of the Atlas to the Occiput

**DOI:** 10.1155/2019/2617379

**Published:** 2019-12-16

**Authors:** Celeste Tavolaro, Hector Pulido, Richard Bransford, Carlo Bellabarba

**Affiliations:** Department of Orthopaedics & Sport Medicine, Harborview Medical Center, 325 Ninth Avenue Seattle, WA 98104, USA

## Abstract

Traumatic atlantooccipital dissociation (AOD) is a severe and usually fatal injury. Patients with assimilation of the atlas to the skull are exposed to a higher risk of injury and delay diagnosis due to the abnormal anatomy. We report two cases of acute traumatic craniocervical dislocation in patients with baseline congenital assimilation of the atlas to the skull. Computer tomography (CT) was used to identify the injury. Computer tomography angiography (CTA) showed variations of the vertebral arteries' location on both patients. Assimilation of the atlas was complete in patient one and partial in patient two. Emergent surgical instrumentation and fusion were performed with a very careful and meticulous posterior dissection. As general rule, most of the patients with CCD will undergo occiput to C2 posterior segmental instrumentation and fusion. In the presented cases, a more extensive fusion was necessary based on the type and severity of the CCJ injury and the anatomical anomalies associated. Postoperatively, patient one remained neurologically intact and patient two died. Alternative fixation techniques should be used to minimize risk of VA injury during the surgical procedures.

## 1. Introduction

Traumatic atlantooccipital dissociation (AOD) is a severe injury of the osseous and ligamentous complex that stabilizes the skull base to C1. The most common mechanism of injury is a high-energy trauma. AOD is associated with brainstem injury, neurogenic shock, and respiratory failure. Diagnosis delay is associated with higher rates of morbidity and mortality [1–4].

Congenital assimilation of the atlas is caused by failure of segmentation between the last occipital and first cervical sclerotome during the early fetal development with a reported incidence of 0.75%-3% [5, 6]. Due to the anomalous craniocervical anatomy, these patients are exposed to a higher risk for delay of injury recognition. A number of anatomical variants are associated with occipitalization of the atlas to the skull such as abnormal location of the vertebral arteries. Treatment options may vary depending on the type and severity of the AOD injury and the anatomical anomalies associated.

We describe two cases of traumatic craniocervical dislocation in patients with baseline congenital assimilation of the atlas to the skull. Our purpose is to highlight the surgical relevance and implications of these traumatic unstable injuries on patients with this abnormal anatomy.

## 2. Case 1

A 53-year-old male was involved in a mountain bike accident. Initial vital signs and neurological examination were normal, but he reported pain and tenderness on the upper part of his neck.

**Figure 1:**
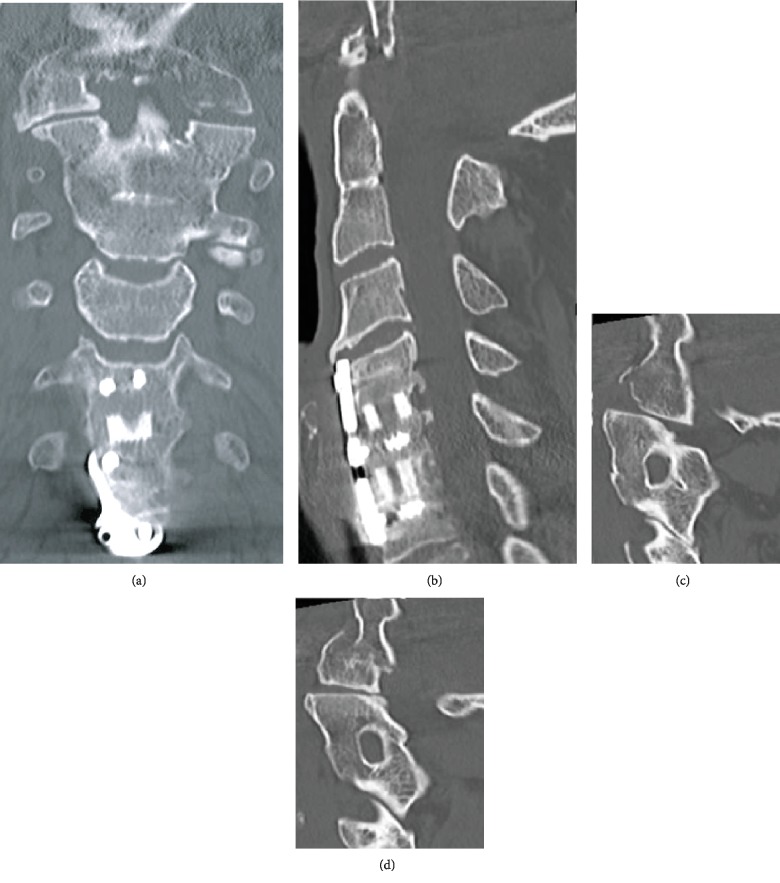
Case 1: cervical CT. (a) Complete C1 assimilation, C2-C3 congenital fusion block. (b) Previous C5-C7 ACDF. The C1 anterior ring is fracture displaced and positioned anterior to the clivus. The C1 posterior arch is fused to the occiput. (c) Left atlantoaxial subluxation. (d) Right condyle C1 lateral mass fracture dislocation.

Cervical computed tomography (CT) revealed evidence of prior C5-C7 ACDF, as well as a complete congenital assimilation between the occiput and the atlas (C1), and also a congenital fusion through the disc space of the C2 and C3 (Klippel Feil syndrome). Imaging demonstrated a comminuted fracture of the anterior arch of C1 extending into the bilateral lateral mases of C1 as well as a fracture extending into the right atlantooccipital fusion mass. There was also distraction on the left side between the atlantoaxial joint (Figure 1). Cervical computed tomography angiography (CTA) showed in the right vertebral artery, as it courses through the fracture occipito-C1 posterior arch assimilation, a focal luminal narrowing of 50% approximately (Biffl type 2) caused by blunt trauma mechanism.

**Figure 2:**
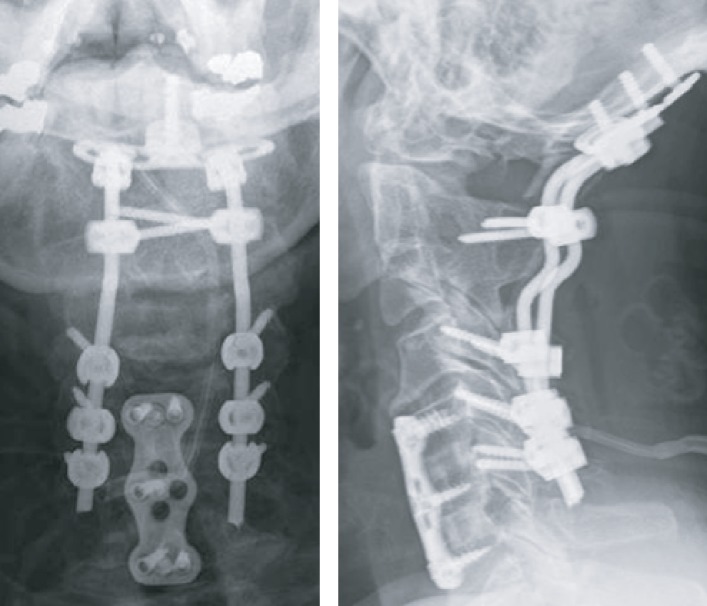
Case 1: postoperative X-ray. Occipito-C6 PSIF.

**Figure 3:**
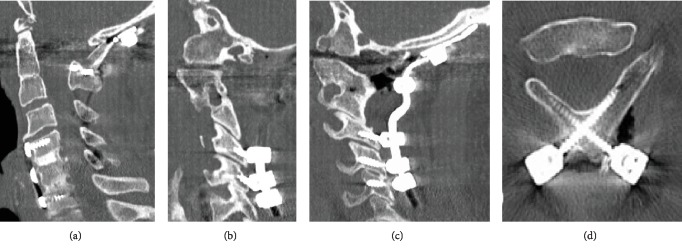
Case 1: postoperative CT. (a) The reduction of the cortical step-off between the anterior arch of C1 and the clivus with partial reduction of the odontoid. (b) Right and (c) left. C1 screws were not placed. The vertebral arteries course bilaterally below the C1 ring. Lateral mass screw placed bilaterally at C4, C5, and C6. (d) Fixation to C2-C3 vertebrae block was obtained by placing laminar screws bilaterally at C2.

Due to the complexity of the case, the patient was transported to our hospital's level 1 trauma center. After obtaining baseline electrodiagnostic signals, Mayfield tongs were applied and the patient was transferred to the Jackson table and rotated into a prone position. A cervical spine lateral fluoroscopic image was performed to evaluate the patient's craniocervical alignment. Electrodiagnostic signals remained stable after positioning. A midline posterior cervical incision and meticulous subperiosteal dissection were performed of the occiput to the level of C6. Open reduction of his craniocervical dissociation and occiput to C6 posterior segmental arthrodesis with placement of cortical screws in the occiput, laminar screws bilaterally at C2, and lateral mass screws bilaterally at C4, C5, and C6 (Figure 2) were performed. Given the fact that the patient had a congenital fusion down to C3 as well as degenerative changes at C3-C4 and C4-C5, and previous ACDF at C5-C6 and C6-C7, his posterior cervical fixation was performed down into his subaxial cervical fusion in order to avoid short-term adjacent segment degeneration at C3-4 and C4-5 (Figure 3). Intraoperative, extensive instability and hypermobility at the C1-C2 levels as well as the C3 to C5 levels were noticeable. The vertebral arteries were identified coursing bilaterally below the C1 ring, given that the posterior ring of C1 was completely coalesced to the occiput. Due to these anatomical findings associated with the right atlantooccipital fracture dislocation, the C1 lateral mass screws were not placed. Atricortical structural iliac crest allograft was applied from the occiput to the C2 spinous process, and locally harvested autologous bone graft, allograft, and tricalcium phosphate bone graft substitutes were placed locally after decorticated the posterior occipitocervical spine.

## 3. Case 2

A 35-year-old male was a restrained driver involved in a head-on high-speed motor vehicle collision. He was unresponsive at the scene, intubated, and transferred to our hospital. On arrival to the emergency room, the patient received a high mechanism work-up which was negative for thoracic, abdominal, or pelvic injuries but did reveal an open right ulna fracture.

**Figure 4:**
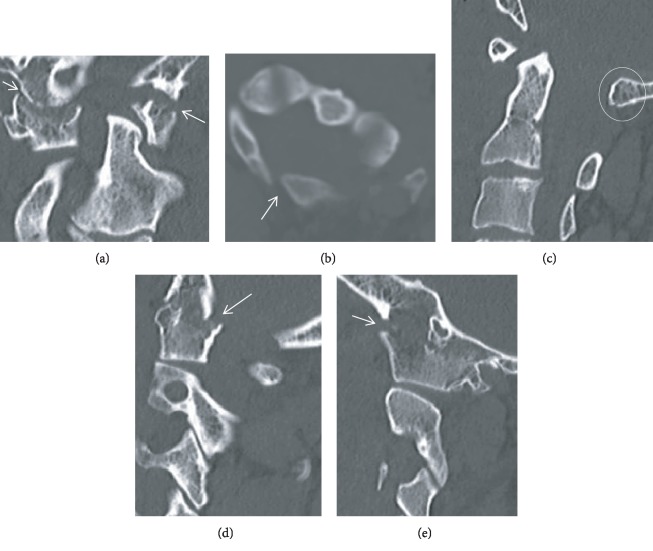
Case 2: cervical CT. C1 congenital partial assimilation to the skull base. (a) The right C1 lateral mass is partially fused with the right occipital condyle. The left C1 lateral mass is completely fused to the left condyle. The tip of the dens is dysplastic and asymmetrically positioned towards the left lateral mass. (b) The rachischisis of the C1 posterior arch. (d, e) Acute fracture dislocation of the previously right and left lateral mass assimilation, respectively.

Head CT showed a left frontal subdural hematoma and epidural hematoma in the upper cervical region anterior to the cord which extended to the C4 level. Cervical CT revealed a congenital partial fusion of C1 to the occiput, involving complete fusion of the left lateral mass with the occipital condyle and partial fusion of the right lateral mass with the right occipital condyle. Posteriorly, the lamina was fused to the occiput on the left side. Craniocervical dissociation due to an acute fracture with distraction through the fused left occipital condyle-C1 lateral mass was recognized. The right C1 lateral mass was laterally subluxated in relation to the C2 lateral mass. The dysplastic tip of the dens showed an asymmetric mild widening on the right side in relation to the C1 lateral mass (Figure 4). Cervical computed tomography angiography (CTA) did not reveal any vascular injury.

Magnetic resonance imaging (T2 sequence) revealed a posterior longitudinal ligament disruption along with the apical, right alar, and right transverse ligaments as well as the disruption of the interspinous ligament at C1-C2 and the ligamenta nuchae. Posterior to the dens, an epidural hematoma that compressed the spinal cord was identified without evidence of spinal cord signal change. Also, a prevertebral hematoma extending from the CCJ to the C6 level was found.

**Figure 5:**
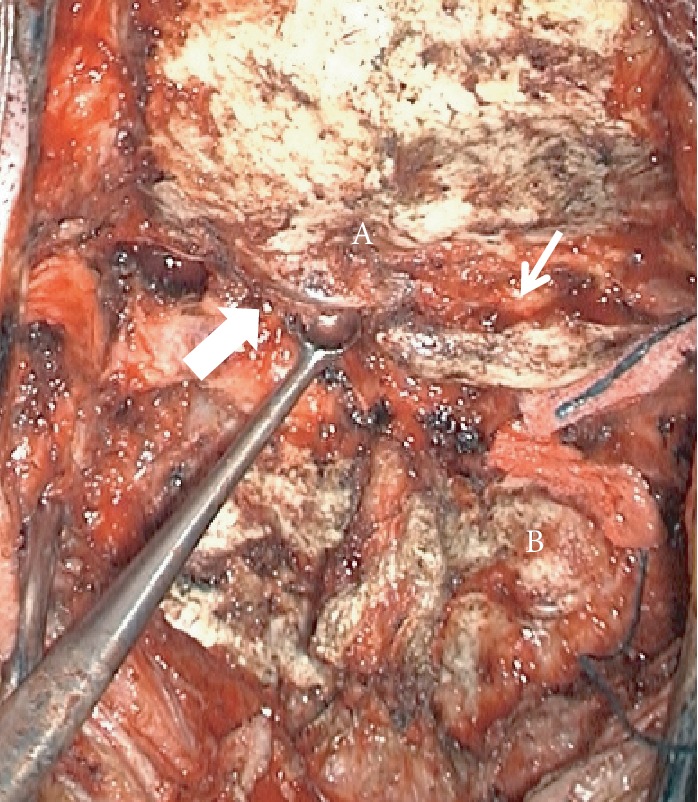
Case 2: intraoperative image. (A) Occiput. (B) C2 vertebra. (Upper arrow) left VA coursing above the free C1 posterior ring. (Thick arrow) right VA fused C1 posterior ring assimilated to the occiput.

**Figure 6:**
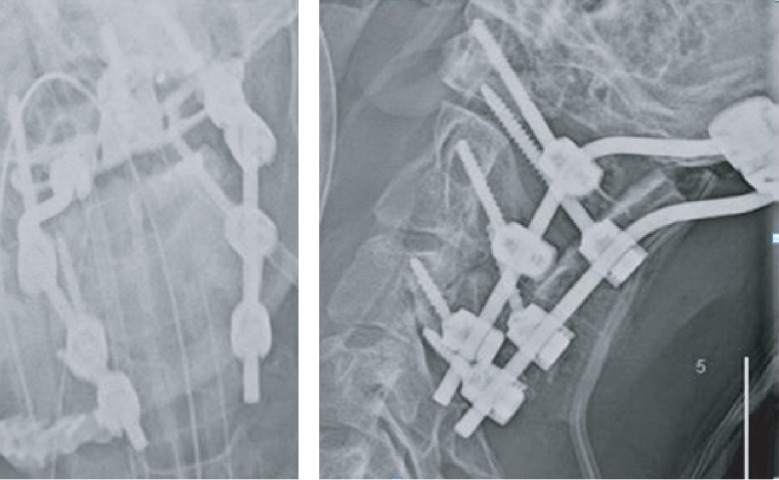
Case 2: postoperative X-ray. Occipito-C6 PSIF.

**Figure 7:**
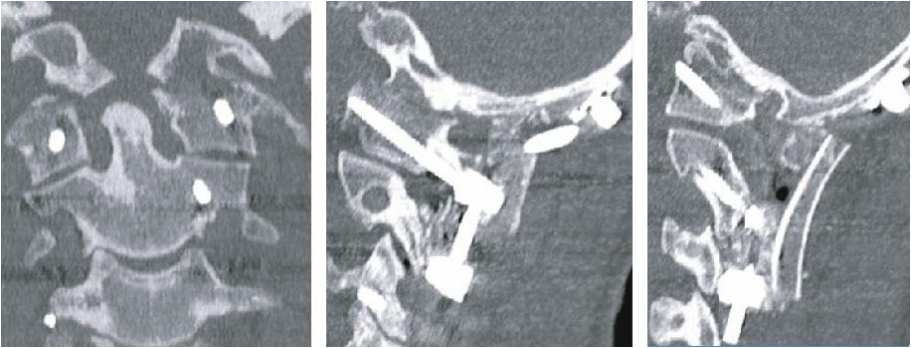
Case 2: CT postoperative. Anatomic alignment with reduction of the atlantooccipital fracture dislocation with bilateral C1, C2, and C3 lateral mass screws.

Initially, the patient underwent a left-sided decompressive craniectomy. Once he was clear for spine surgery, baseline electrodiagnostic signals were obtained. Mayfield tongs were applied, and the patient was transferred to the Jackson table and rotated into a prone position. A cervical spine lateral fluoroscopic image was performed to evaluate the patient's craniocervical alignment. Electrodiagnostic signals remained stable after positioning. A midline posterior cervical incision and dissection were performed from the occiput to C3. Open reduction of the craniocervical dislocation and occiput to C3 posterior segmental arthrodesis was performed. C1 and C3 bilateral lateral mass screws and bilateral C2 pars screws were placed. Intraoperatively, significant instability between the occipital to C2 region was noticed as well as an abnormal vertebral artery (Figure 5). On the left side, the vertebral artery traversed underneath the fused occiput-C1 ring, whereas, on the right side, the C1 posterior arch was free of the occiput and the vertebral artery traversed above this (Figures 6 and 7). Atricortical structural iliac crest allograft was applied from the occiput to the C2 spinous process, and locally harvested autologous bone graft, allograft, and tricalcium phosphate bone graft substitutes were placed locally after decorticated the posterior occipitocervical spine.

During the postoperative hospital stay, the patient had a complication related to his severe traumatic brain injury which led to death.

## 4. Discussion

Traumatic atlantooccipital dissociation is a rarely observed but potentially severe injury of the osseous and ligamentous complex that stabilizes the skull base to the spine. The most common mechanism of injury is a high-energy trauma. The delay of injury recognition is associated with higher rates of morbidity and mortality [1–4]. In our patients, the atlantooccipital injury was recognized with index CT imaging.

Congenital assimilation of the atlas is caused by failure of segmentation between the last occipital and first cervical sclerotome during the early fetal development [5, 6]. It is described in the literature as the most common anomaly of the craniocervical junction with an incidence of 0.75%-3% and a male to female ratio of 5 : 1 [7–9]. Complete and partial assimilation have been described. Kim et al. [10] classified the assimilation of the atlas in zones depending on the location of the bony fusion between the atlas and the occiput. Zone 1 fusion involves the anterior arch of the atlas in front of the lateral masses; zone 2 fusion primarily involves the lateral processes; zone 3 fusion involves the posterior arch of the atlas behind the lateral masses; and zone 4 involves a combination of zones. According to this classification, the type of assimilation in both our patients included a combination of zones or zone 4. In case 1, the patient presents a complete assimilation of the atlas to the occiput, including the fusion of the bilateral lateral masses plus the anterior and posterior arch to the occiput. In case 2, the assimilation of the atlas was partial, including the complete fusion of the left lateral mass and the left side of the posterior arch of C1 to the skull and a partial fusion of the right lateral mass with the right occipital condyle.

There are a number of anatomical variants associated with occipitalization of the atlas to the occiput which are essential to know to make a correct diagnosis and surgical decision in the setting of trauma to the craniocervical junction (CCJ). Some of these anatomical variants can mimic fractures and should not be misinterpreted in patients with craniocervical injuries. Condylus tertius and calcification of the alar ligament may mimic an avulsion fracture of the occipital condyle. The posterior or/and anterior atlas rachischisis could be confuse with a C1 ring fracture. The ossiculum terminale may mimic a type 1 dens fracture and the Os odontoideum, a type 3 dens fracture [5]. The patient in case 2 presented a partial posterior ring assimilation associated to a posterior ring rachischisis, as well as a dysplastic C2 dens.

Furthermore, the assimilation of the atlas has been associated with other craniovertebral anomalies such as basilar invagination, atlantoaxial subluxation, variations of the vertebral arteries' (VAs') location, and cervical vertebral fusions [11]. The presence of blocked vertebrae between the skull and the atlas may accelerate the development of atlantoaxial instability, due to compensatory motion at the atlantoaxial joint. Both cases did not show any signs of basilar invagination or atlantoaxial subluxation. However, as can occur in up to 70% of the cases with assimilation of the atlas [8], the patient in case 1 presented a fusion block of the second and third cervical vertebrae (Klippel Feil syndrome).

In patients without anatomical variants of the craniocervical junction, the path of the vertebral artery (VA) is described in four segments: the first segment originates from the subclavical artery and enters the C6 transverse process, the second segment courses from C6 to C2 transverse process, the third from C2 to the foramen magnum, and the fourth segment enters the cranium to attend the basilar artery [12]. Patients with assimilation of the atlas may demonstrate an abnormal course of VA at the third segment, due to the osseous fusion between the atlas and occiput. Four types of VA courses were described in patients with assimilation of the atlas [13]. Type I VA is wherein the vessel enters the spinal canal below the C1 posterior arc and courses below the occipitalized C1 lateral mass; type II wherein the VA courses on the posterior surface of the occipitalized C1 lateral mass or makes a curve on it; type III in which the VA ascends laterally after leaving the axis transverse foramen, enters an osseous foramen created between the atlas and occipital bone, then reaches the cranium; and type IV wherein the VA is absent.

Both of our patients proved to have an abnormal course of the VA at the third segment. In case 1, the VA was identified as coursing bilaterally below the C1 ring, given that the posterior ring of C1 was completely coalesced to the occiput, corresponding to type I of the VA course classification described above. In case 2, on the left side, where a partial fusion of C1 was present, the VA coursed below the assimilated C1, and on the right side, where the C1 ring was free, the VA had a normal course. In this patient, there was no correlation with any of the VA course classifications.

As general rule, most of the patients with CCD will undergo at least an occiput to C2 posterior segmental instrumentation and fusion. Treatment options may vary depending on the type and severity of the CCJ injury and the anatomical anomalies associated. A detailed CT and CTA review should be performed in order to have a better understanding of the injury and the potential anatomical variations, especially to determine the course of the vertebral arteries. A very careful and meticulous posterior dissection should be performed in all the cases with CCD but especially in those with bony assimilations and VA abnormalities, in order to decrease the risk of VA injury during the surgical approach [11]. When the VA courses above the posterior C1 arch, the placement of lateral mass screws at C1 is relatively safe. However, the risk may increase significantly with any anomalous coursing of the VA, and other techniques of internal fixation may be safer options [10].

In both cases presented in this study, given the instability, the treatment of choice was open reduction and occipitocervical fusion. However, in case 1, it was not possible to place the C1 lateral mass screws due to the anomalous path of the VA, along with the fracture of the hypoplastic C1 lateral mass-condyle. The C2-C3 block vertebrae associated with an anomalous lateral mass did not allow the placement of lateral mass screws at these levels, and thus the fixation was obtained by the placement of C2 bilateral laminar screws. Finally, due to the previous C5-C7 ACDF associated with intraoperative findings of extensive instability and hypermobility at C3-C4 and C4-C5 levels, the extension of the instrumentation was performed down to the C6 level. In case 2, intraoperative, the dislocation between the occipital and C2 was noticed. On the right side where the C1 posterior arch was not assimilated to the occiput, the VA traversed above this, allowing for safe placement of the C1 lateral mass screw. On the left side, the VA was below and well protected by the ring attached to the occiput, allowing the placement of the C1 lateral mass. The patients had smaller size of C2 pedicles, particularly on the right side for screw placement. Ji et al. [14] describe in their study that the C2 pars/pedicle architectures are significantly smaller in patients with assimilation of the atlas than in the normal population. They suggested that translaminar screw is the more viable option in comparison with pedicle screw for C2. In our case, short screws were placed at C2 level but we do not consider that it had sufficient stability given the injury, and thus, we decided to extend to the fixation down to the C3 level with lateral mass screws.

## 5. Conclusion

- High clinical suspicion, early recognition, and treatment of traumatic craniocervical dissociation are associated with better outcomes and a higher survival rate
- Patients with assimilation of the atlas to the skull frequently have other associated craniocervical malformations, such as variations of the vertebral arteries' location and other cervical vertebral fusions
- The identification of an abnormal path of the vertebral artery, under the posterior ring of C1, along a C1 lateral mass hypoplastic and fracture, made the C1 lateral mass screw placement difficult and dangerous. Alternative fixation techniques should be used to minimize risk of VA injury during the surgical procedures
- The association of cranial cervical assimilations with other subaxial congenital fusions may require a more extensive instrumentation and fusion than what is normally performed in cases without assimilation of the atlas to the occiput
